# Considerations for the Optimal Timing, Duration, Frequency, and Length of an Intermittent Fasting Regimen for Health Improvement

**DOI:** 10.3390/nu12092567

**Published:** 2020-08-25

**Authors:** Benjamin D. Horne

**Affiliations:** 1 Intermountain Medical Center Heart Institute, Salt Lake City, UT 84107, USA; benjamin.horne@imail.org; Tel.: +1-801-507-4708; Fax: +1-801-507-4792; 2 Division of Cardiovascular Medicine, Department of Medicine, Stanford University, Stanford, CA 94063, USA

Newer, better approaches to weight loss continue to spark the interest of vast numbers of people, especially in Western nations, as they search out solutions to increasingly elevated rates of obesity, the health consequences of that obesity, and the often unspoken but financially prescient cosmetic implications of obesity that drive the billion-dollar weight loss and diet control industry. Recently, though, intermittent fasting—the simple approach of abstaining from food, which is typically a low-cost behavior—has dramatically risen in popularity as a method for both weight management and health improvement. A limited number of well-conducted randomized controlled trials of intermittent fasting with >50 participants and parallel control arms show that intermittent fasting diets reduce weight as effectively as standard caloric restriction diets [1,2,3]. While weight loss through caloric restriction is well-established to provide health improvements such as reductions in blood pressure and cholesterol levels that are secondary to weight loss, it also provides weight-independent benefits to health [4]. Similarly, intermittent fasting provides health benefits that arise due to weight loss and through mechanisms that act regardless of weight change [5]. The optimal intermittent fasting regimen for weight loss and for health improvements, however, remains unknown today.

Intermittent fasting is a general term encompassing a variety of dietary regimens that vary based on the duration, frequency, timing, and length of fasting. While sometimes referred to as a lifestyle, intermittent fasting is usually practiced as a diet that leads to weight loss. Some regimens are attuned to rapid weight loss, including popularized versions such as alternate-day fasting [1], and twice-weekly fasting [2,3] that utilize a duration of about one day in each fasting episode and a frequency of more than once per week. These regimens employ higher frequency and duration than other fasting methods, but the timing of fasting is not specified. Further, they may be difficult to maintain over the length of a year or more, as evidenced by substantial drop-out rates in controlled trials [1,2,3], which may bode poorly for fasting in actual-practice situations.

Other intermittent fasting regimens include early time-restricted feeding (TRF) in which daily fasting of 12–21 h is conducted, most commonly for about 16 h following a late lunch/early dinner (around 3:00pm) and ending the next morning at breakfast (around 7:00am) [5,6]. TRF explicitly integrates timing considerations and is high frequency (daily), but is lower duration than most fasting regimens (lasting for fewer than 24 h per episode). Other fasting regimens include 10-day juice or bone-broth fasts, and periodic fasting of a duration of one day or more on a frequency of once every 3–4 weeks. Additionally, religious fasting practices can be included which, while outside of some weight loss-centric definitions, comfortably fit into the term “intermittent” fasting because of their irregular non-continuous patterns, including both periodic fasting [7], and Ramadan that is late TRF in which fasting ends late in the day (instead of at breakfast as it does in the early TRF approach) [8].

Randomized trials show that both a 24-h fast and a TRF (≈16 h) fast result in important biological changes in humans that should lead to extensive health improvements such as chronic disease prevention when practiced repeatedly over a length of years [9,10,11,12,13,14,15,16]. For example, a 24-h fast reduced the microbiome-related cardiac risk factor, trimethylamine N-oxide [10] and induced various changes potentially related to autophagy and energy management [9,10]. However, the 24-h fast also increased red blood cell parameters such as hematocrit and hemoglobin [9], while a TRF fast reportedly decreased hematocrit and white blood cell parameters [12]. In another study, early TRF showed improvement in fasting glucose compared to late TRF, but timing of TRF initiation did not affect glycemic response to a test meal in the same study [14].

Such variations in duration, frequency, timing, and length of fasting may have different effects, including that one or multiple regimens may lead to optimal weight loss compared to other fasting regimens. Further, a different regimen or different set of regimens may be more beneficial for preventing or resolving health concerns, such as preventing chronic cardiometabolic diseases and ameliorating the risk factors for such diseases, although it does not lead to as much weight loss. For example, because weight loss-independent benefits may arise from fasting [5], whether the weight loss fasting regimens (e.g., [1,2,3]) are the optimal ones for achieving cardiometabolic benefits not related to weight loss is unknown. The length of adherence to a fasting regimen is also little studied, partly due to the length of years needed to observe reductions in risk of chronic diseases such as diabetes and coronary artery disease, which develop over periods as long as 20–40 years [7]. Studies with larger sample sizes and longer-lengths of regimen participation are needed to study clinical outcomes of intermittent fasting. Finally, both early and late TRF have key differences compared to other forms of fasting [5,6] and compared to each other [6,8] of which the timing of initiating and ending fasting during the day may be significant.

To these points, Paoli and colleagues reviewed the potential effects of different frequencies and timing of meals and fasting on weight loss and the cardiovascular system, specifically focusing on the role of TRF in human health [6]. A crucial feature of their article is to cut through the haze of apparently conflicting findings regarding the frequency and timing of meals that have arisen over the last half century in which different study designs have produced discordant results for higher vs. lower frequency of meals and with regard to when meals are consumed. They especially examine the critical juncture of breakfast and the effects of caloric intake into the late hours of the evening [6]. Many of the studies previously reporting that fewer meals per day is associated with higher risk of coronary heart disease and greater risk of weight gain were cross-sectional designs, limiting their generalizability and interpretation of causality [6]. Further, infrequent meals or an irregular pattern of meals may be markers of other behaviors that lead to weight gain. The simple counting of the number of meals may overlook varied patterns in the meals that were not characterized by studies historically [6]. This includes the potential that these differing patterns of meals may capture information related to the types of macronutrients that are consumed.

Paoli also thoroughly addresses the issue of breakfast. The eating versus skipping of breakfast is a point of conflicting evidence in the literature, with some studies using retrospective or cross-sectional designs being those that indicate that skipping breakfast leads to better health [6]. However, prospective cohort studies contradict those findings, instead indicating that individuals who consistently consume breakfast have lower risk of poor health [6]. If anything, the best data suggest that skipping dinner is most beneficial for cardiometabolic health [6]. This may be in part because people who eat dinner also are more likely to consume additional calories after dinner and late into the evening.

Timing of meals, thus, may have critical implications for metabolic health through mechanisms of glycemic control and via circadian clock mechanisms. Specifically, greater consumption of calories in the early part of the day appears to lead to better cardiac health and lower weight, likely in part because the body’s circadian clock influences glycemic control and cholesterol metabolism [6].

In this context, the potential merits of TRF are proposed because of both the connection to fasting as well as the explicit integration of the timing of eating and fasting that is a component of the early TRF regimen [6]. Various physiological mechanisms may be in play through fasting generally, and TRF specifically, including stimulation of autophagy and induction of fat metabolism. The similarities and differences of a caloric restriction diet, the ketogenic diet, and a fasting diet are considered by Paoli, indicating many co-incident physiological influences of these three distinct dietary approaches [6]. Whether TRF produces the vast array of potential benefits in humans that animal models suggest that it might remains to be seen given that the few published randomized controlled trials of TRF are primarily small pilot studies of <50 participants [11,12,13,14,15], with one larger trial examining alternate-day fasting versus TRF and comparing these to controls [16].

The Paoli review concludes that several complex biological processes are involved in meal- and fasting-related determinants of human health [6]. Circadian clock-related mechanisms and time-dependent variations in macronutrient intake are crucial among these considerations. The authors suggest that “A regular meal pattern including breakfast consumption, consuming a higher proportion of energy early in the day, reduced meal frequency (i.e., 2–3 meals/day), and regular fasting periods may provide physiological benefits...” [6] and that TRF may be an important feature in achieving those benefits.

In summary, good evidence exists for the cardiovascular and metabolic benefits of intermittent fasting due to the unique frequency and timing characteristics of fasting regimens, such as TRF. These benefits may arise from weight loss and other physiological mechanisms. Intermittent fasting is generally safe in apparently healthy individuals, usually causing minor adverse events such as hunger, fatigue, and headache. For people with diagnosed chronic diseases, though, such as type 2 diabetes and coronary artery disease, safety of intermittent fasting is not trivial and must be carefully considered by those evaluating whether to engage in fasting for health purposes [17]. Overall, evidence is accumulating that intermittent fasting is an effective and safe method of weight loss and a tool for chronic disease prevention. Further investigation into the intermittent fasting mechanisms of benefit in humans and the resulting clinical outcomes should be conducted.
